# Controlled Ascent Rate Enhances Autophagy and Mitigates Acute Lung Injury in Rats Exposed to High‐Altitude Hypoxia by Inhibiting Oxidative Stress and Inflammation

**DOI:** 10.1002/gch2.202400362

**Published:** 2025-04-27

**Authors:** Kairui Huang, Wenhui Shi, Jiajia Li, Xiaoyan Ma, Jiangwei Liu

**Affiliations:** ^1^ Department of Graduate School Xinjiang Medical University Urumqi Xinjiang Uygur Autonomous Region 830000 China; ^2^ Key Laboratory of Special Environmental Medicine of Xinjiang General Hospital of Xinjiang Military Command Urumqi Xinjiang Uygur Autonomous Region 830000 China; ^3^ Xinjiang Medical University Sixth Affiliated Hospital Urumqi Xinjiang Uygur Autonomous Region 830000 China

**Keywords:** acute lung injury, autophagy, high‐altitude hypoxia, inflammation, oxidative stress

## Abstract

The gradual ascent strategy, an effective measure to prevent acute mountain sickness by enabling the body to adapt to high–altitude hypoxia, has an unclear mechanism. This study explores controlled ascent rates' effects on autophagy, oxidative stress, inflammation, and lung injury in rats exposed to high‐altitude hypoxia, hypothesizing that gradual ascent can activate autophagy, reduce oxidative stress and inflammation, and improve lung injury. 70 male Sprague‐Dawley rats are divided into seven groups, including a normal control group and high‐altitude hypoxia for 24, 72, and 120 h, with or without controlled ascent rates. Lung tissues are analyzed for the wet‐to‐dry weight ratio, histopathology, inflammatory cytokines, oxidative stress markers, and autophagy‐related protein expression. Results show that controlled ascent rates reduced lung injury, oxidative stress, and inflammation in rats exposed to high‐altitude hypoxia while increasing autophagy. This study indicates that gradual ascent can be an effective strategy for reducing lung injury in high‐altitude areas by regulating autophagy and reducing oxidative stress and inflammation.

## Graphical abstract

This study explores how gradual ascent rates reduce acute pulmonary injury in rats exposed to high‐altitude hypoxia by activating autophagy and suppressing oxidative stress and inflammation. Results show that controlled ascent effectively mitigates such injury, highlighting its potential as a preventive strategy.

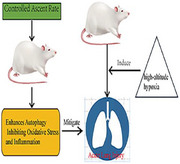

## 1 Introduction

When individuals ascend to altitudes of 2500 meters or higher, they may encounter an environment characterized by low atmospheric pressure and oxygen deficiency, which poses a significant challenge to the body. If the body fails to adapt to these conditions, tissue hypoxia may occur, a state where there is an insufficient supply of oxygen. This can lead to a range of serious health issues collectively known as altitude sickness.^[^ ^1^ ^]^ Lung tissue and function may be compromised in such an environment. Pulmonary issues may arise including interstitial congestion, inflammatory cell infiltration, thickening of the alveolar walls, and alveolar edema. Severe cases can lead to high‐altitude pulmonary edema, ultimately resulting in respiratory failure and death.^[^ ^2^ ^]^ Previous studies have indicated that oxidative stress can be rapidly triggered in certain tissues, such as the lungs, following exposure to high‐altitude hypoxia. This can ultimately lead to structural alterations in lipids, proteins, and DNA, resulting in lung cell damage.^[^ ^3^ ^]^ Therefore, reducing the production of pro‐inflammatory cytokines and decreasing the levels of oxidative stress can effectively alleviate acute hypoxic lung injury in rats.^[^ ^4^ ^]^ Controlling the ascent rate is an effective method to prevent acute mountain sickness and mitigate pulmonary damage caused by hypobaric hypoxia at high altitudes.^[^ ^5^ ^]^ At present, the mechanism by which gradual ascent in altitude can prevent acute organ damage in a hypobaric hypoxic environment at high altitudes remains unclear. However, gradual ascent to high altitudes can also be considered an effective method of hypoxia preconditioning. A substantial body of research has found that low‐dose hypobaric hypoxia preconditioning can mitigate lung injury induced by exposure to low‐pressure hypoxia by reducing the infiltration of macrophages in lung tissue and decreasing the expression of pro‐inflammatory cytokines.^[^ ^6^ ^]^

In the hypoxic environment of high altitude, organ damage is attributed to acute exposure to high altitude/hypoxia, which increases the production of reactive oxygen species (ROS), leading to oxidative stress. The escalation of ROS further injures cellular structures and is associated with the progression of high‐altitude illnesses (HAIs).^[^ ^7^ ^]^ Autophagy is a critical cellular defense mechanism against oxidative stress, and research has discovered that the interplay between autophagy and ROS plays an essential role in the development and progression of pulmonary diseases.^[^ ^8^ ^]^ Autophagy is a cellular process that maintains and regulates intracellular homeostasis by forming lysosomes to degrade and recycle cellular components.^[^ ^9^ ^]^ Autophagy plays a crucial role in lung injury, responding to oxidative stress induced by hypoxia and maintaining intracellular homeostasis by removing damaged proteins and mitochondria,^[^ ^10^, ^11^ ^]^ The specific mechanisms include ROS activating autophagy by influencing multiple signaling pathways, promoting cellular adaptation, and reducing oxidative damage,^[^ ^12^, ^13^ ^]^ However, autophagy is not always beneficial to the organism; appropriate activation of autophagy can assist the body in adapting to the environment, while excessive autophagy is detrimental, leading to cell death and exacerbating organ damage.^[^ ^14^ ^]^

Although a controlled‐ascent helps humans prevent acute mountain sickness and reduces high‐altitude hypobaric hypoxia‐induced lung injury, it remains unverified in rats. Therefore, our study hypothesizes that gradual ascent in altitude can activate autophagy and regulate autophagy levels, ultimately reducing oxidative stress and inflammation to ameliorate high‐altitude hypoxia‐induced rat lung injury.

## 2 Results

### 2.1 Morphological Changes of Lung Tissue

**Figure 1:**
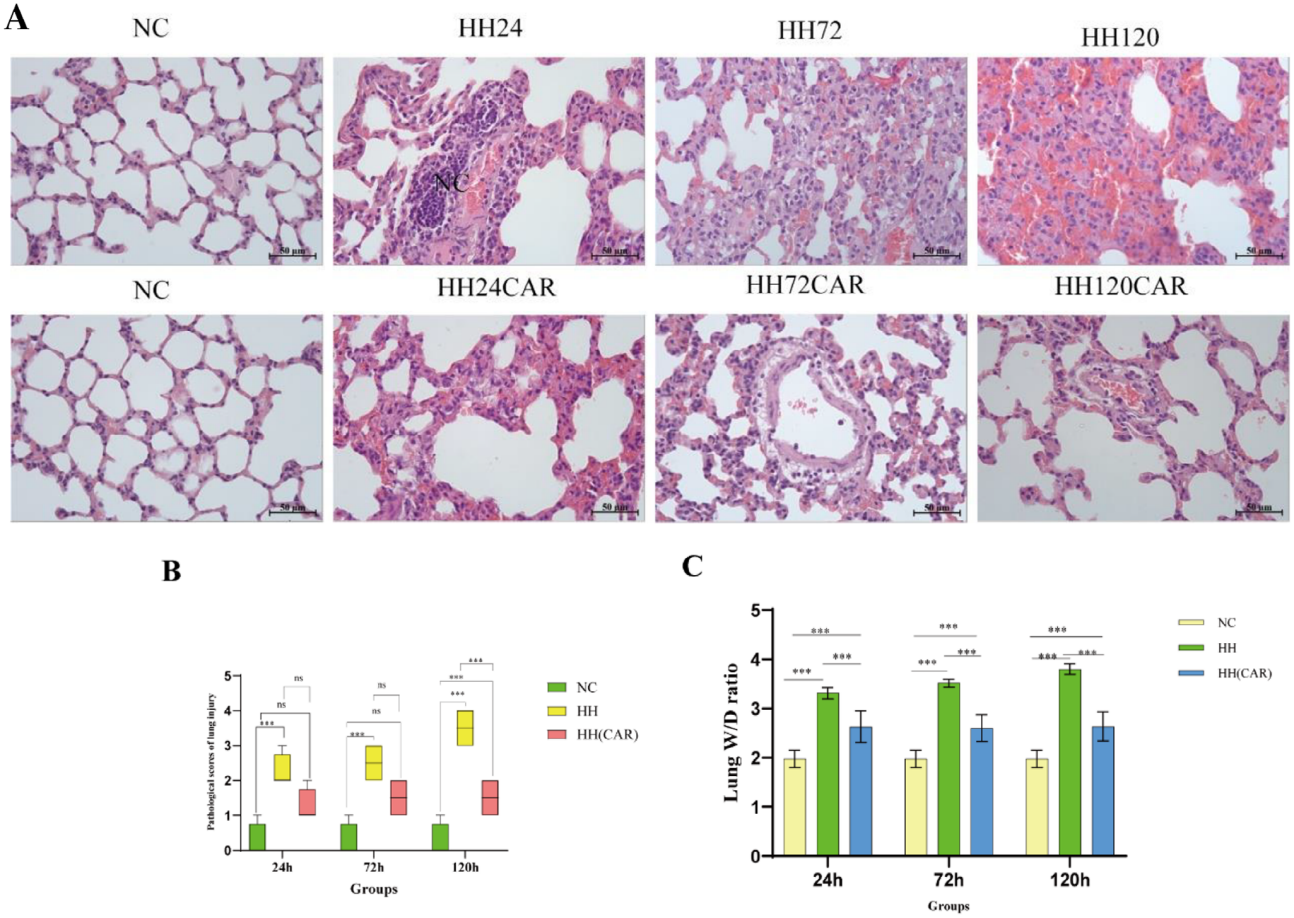
Histopathological assessment and scoring of Acute Lung Injury (ALI) under an optical microscope(40 × 10). A) Representative histopathological images of lung tissue from each experimental group. B) Pathological scores of lung injury. C) Lung W/D ratio. NC (Normal Control) group, HH (High‐Altitude Hypoxia) group, and HH(CAR)(a group subjected to High‐Altitude Hypoxia after Controlled Ascent Rate). Data are presented as the mean ± SD (*n* = 10). ^*^ *p *<0.05, ^**^ *p *<0.01, ^***^ *p *<0.001.

In the histological assessment of lung injury‐related indices using H&E staining, we observed that the lung tissue structure of the control rats remained normal, with no detectable pathological changes (**Figure** **1**A). In contrast, the lung tissue of the HH group exhibited significant pathological alterations, including interstitial pulmonary edema, increased thickness of alveolar walls, increased neutrophil counts, and pulmonary hemorrhage. Over time, these pathological features further intensified, with severe cases even presenting the typical histological changes of ALI (Figure 1A). However, in the HH (CAR) group, the lung injury in rats was significantly milder, primarily characterized by mild interstitial edema, occasionally accompanied by a small number of inflammatory cell infiltrations. Notably, these injuries showed little variation across different time points (Figure 1A). Acute Lung Injury Pathological scoring analysis further confirmed that the degree of lung injury in the HH group rats was more severe than that in the NC group (*p* < 0.001; Figure 1B). Additionally, the lung injury scores in the HH (CAR) group were lower than those in the HH group at 24, 72, and 120 h, with a significant difference at the 120 h mark (*p* < 0.001). The results of the lung wet‐to‐dry weight ratio (W/D) indicated that compared to the NC group, the lung W/D ratio in the HH group was significantly elevated at each time point (*p* <0.001), while the HH (CAR) group also showed an increase (*p* <0.001), but a significant reduction compared to the HH group (*p* <0.001), as shown in Figure 1C, these results suggest that lung injury in the HH (CAR) group was mitigated compared to that of the HH group.

### 2.2 Inflammatory Factor Levels in Lung Tissue

**Figure 2:**
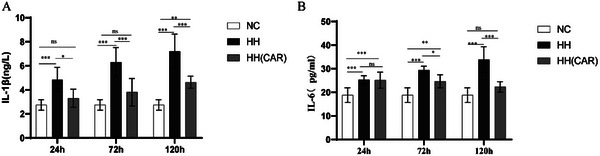
Changes in Inflammatory Factors in Lung Tissue of Rats in Different Groups. A) Interleukin (IL)‐1β in Lung Tissue B) Interleukin IL‐6 in Lung Tissue. NC (Normal Control) group, HH (High‐Altitude Hypoxia) group, HH(CAR)(a group subjected to High‐Altitude Hypoxia after Controlled Ascent Rate).Data are presented as the mean ± SD (*n* = 10). ^*^ *p *<0.05, ^**^ *p *<0.01, ^***^ *p *<0.001.

Based on the data presented in **Figure** **2**, it can be observed that after 24, 72, or 120 h of hypoxia stress, the expression levels of IL‐1β and IL‐6 in rats were significantly increased compared to the NC group *(p* <0.001). However, in the group treated with HH (CAR), the expression levels of these two cytokines were significantly reduced compared to the hypoxia stress group (HH group) (*p* <0.001). Notably, at 24 and 72 h, the expression levels of IL‐1β and IL‐6 in the HH (CAR) group were not significantly different from those of the NC group (*p* >0.05). These findings suggest that lung injury in the HH (CAR) group was alleviated compared to the untreated HH group.

### 2.3 In the Assessment of Changes in GSH‐Px, SOD, and MDA Levels Among Various Groups of Rats

**Figure 3:**
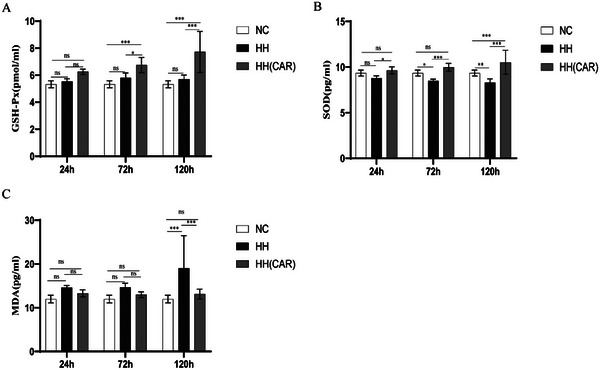
Experimental results of oxidative stress in rats of each group. (A)GSH‐Px, glutathione peroxidase. (B)SOD, superoxide dismutase. (C)MDA, malondialdehyde. NC (Normal Control) group, HH (High‐Altitude Hypoxia) group, HH(CAR)(a group subjected to High‐Altitude Hypoxia after Controlled Ascent Rate).Data are presented as the mean ± SD (*n* = 10). ^*^ *p* <0.05, ^**^ *p *<0.01, ^***^ *p* <0.001.

Based on the data from **Figure** **3**, we observed the following results: In the HH group, at 24 h, the activities of SOD and GSH‐Px, as well as the expression levels of MDA, showed no significant difference compared to the NC group (*p* >0.05). However, as time extended to 72 and 120 h, the activity of SOD in the HH group significantly decreased (*p* <0.01), and the expression levels of MDA significantly increased after 120 h (*p* <0.01). In contrast, in the HH (CAR) group, the activities of SOD and GSH‐Px were significantly higher at 72 and 120 h compared to the HH group (*p* <0.05), and the expression levels of MDA were significantly reduced after 120 h (*p* <0.001). These results suggest that HH (CAR) treatment has a positive effect on maintaining the stability of oxidative stress indicators.

### 2.4 Analysis of Arterial Blood Gas levels

**Figure 4:**
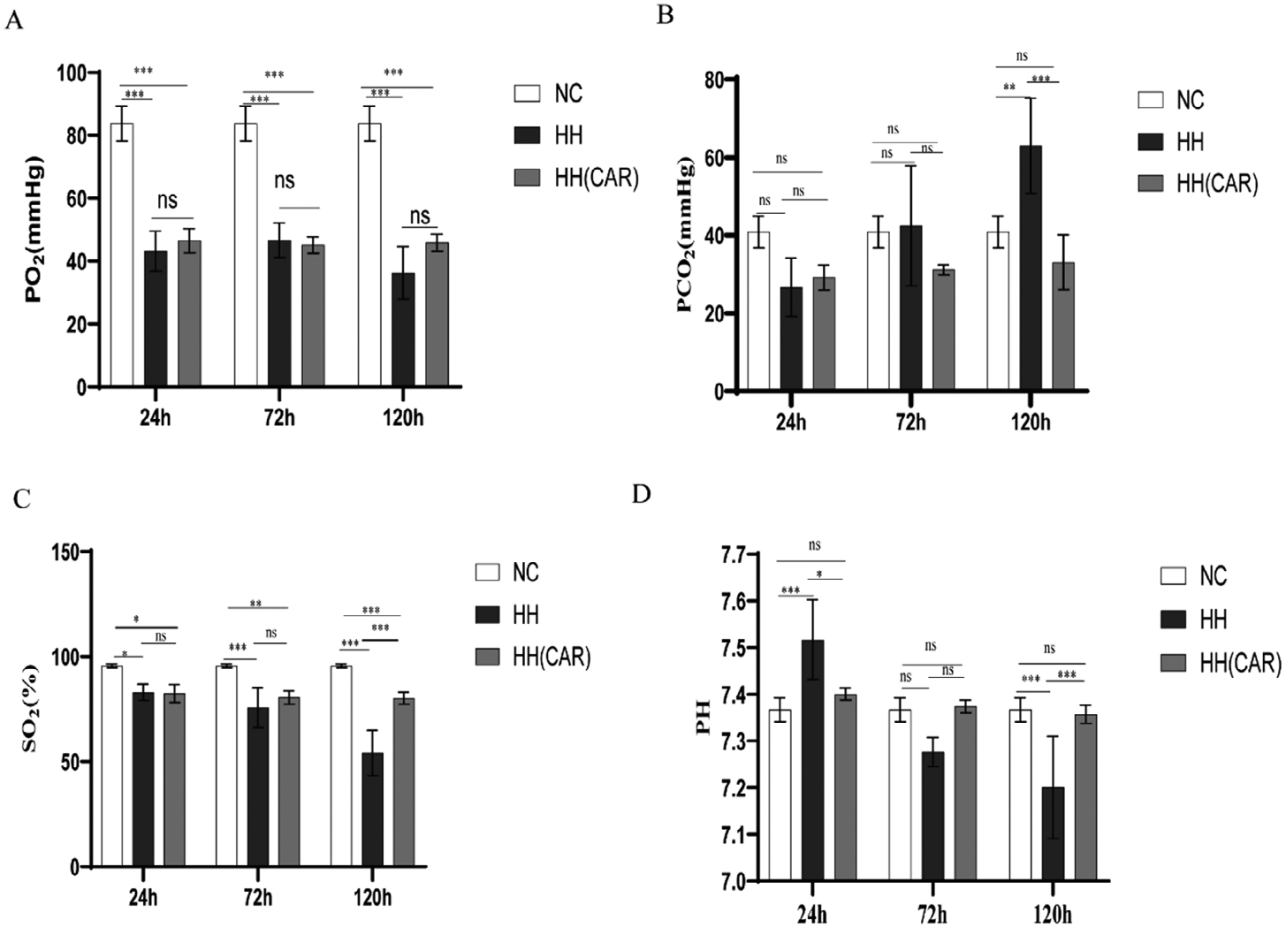
Changes in arterial blood gas indicators in the rats of different groups. A) arterial oxygen partial pressure (PO2), B) carbon dioxide partial pressure (PCO2), C) arterial oxygen saturation (SO2) (D)potential of hydrogen (PH). NC (Normal Control) group, HH (High‐Altitude Hypoxia) group, and HH(CAR)(a group subjected to High‐Altitude Hypoxia after Controlled Ascent Rate). Data are presented as the mean ± SD (*n* = 10). ^*^ *p* <0.05, ^**^ *p *<0.01¸ ^***^ *p* <0.001.

As depicted in **Figure** **4**A–D, compared to the NC group, the partial PO2 in the HH group was significantly reduced at 24, 72, and 120 h (*p* <0.001, Figure 4A). In contrast, no significant differences in PO2 levels were observed between the HH(CAR) group and the HH group at these time points (*p* >0.05, Figure 4A). At the 120 h mark, the PCO2 in the HH group was significantly elevated compared to the NC group (*p* <0.01, Figure 4B), whereas the PCO2 level in the HH(CAR) group was significantly decreased compared to the HH group at 120 h (*p* <0.05, Figure 4B), and did not differ significantly from the NC group. The partial SO2 in the HH group was consistently and significantly lower than that in the NC group at all measured time points (*p* <0.05, Figure 4C), and the SO2 levels in the HH(CAR) group showed a significant increase at 120 h compared to the HH group, which was marked (*p* <0.001, Figure 4C). The pH in the HH group rats was significantly higher than that in the NC group at 24 h (*p* <0.001, Figure 4D), while the pH in the HH(CAR) group was significantly lower than that in the HH group at 24 h (*p* <0.05, Figure 4D), and not significantly different from the NC group. At 120 h, the pH in the HH group was significantly lower than that in the NC group (*p* <0.001, Figure 4D), and the pH in the HH(CAR) group was significantly higher compared to the HH group at 120 h (*p* <0.001, Figure 4D), with no significant difference observed when compared to the NC group. These findings suggest that the HH(CAR) intervention may modulate the respiratory parameters, potentially ameliorating the effects of hypoxia.

### 2.5 Variations in Plasma Lactate Levels

**Figure 5:**
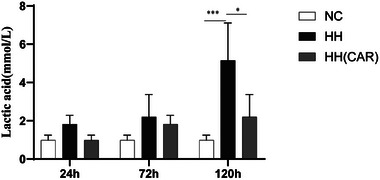
Variations in Plasma Lactate Levels Among Different Groups of Rats. NC (Normal Control) group, HH (High‐Altitude Hypoxia) group, and HH(CAR)(a group subjected to High‐Altitude Hypoxia after Controlled Ascent Rate). Data are presented as the mean ± SD (*n* = 10). ^*^ *p *<0.05, ^**^ *p *<0.01, ^***^ *p* <0.001.

As shown in **Figure** **5**, the lactate level in the HH group was significantly higher than that in the NC group at 120 h (*p* <0.001), while the lactate level in the HH(CAR) group was significantly lower than that in the HC group (*p* <0.05), and showed no significant difference when compared to the NC group.

### 2.6 Changes in the Expression of Autophagy‐Related Proteins in Lung Tissue

**Figure 6:**
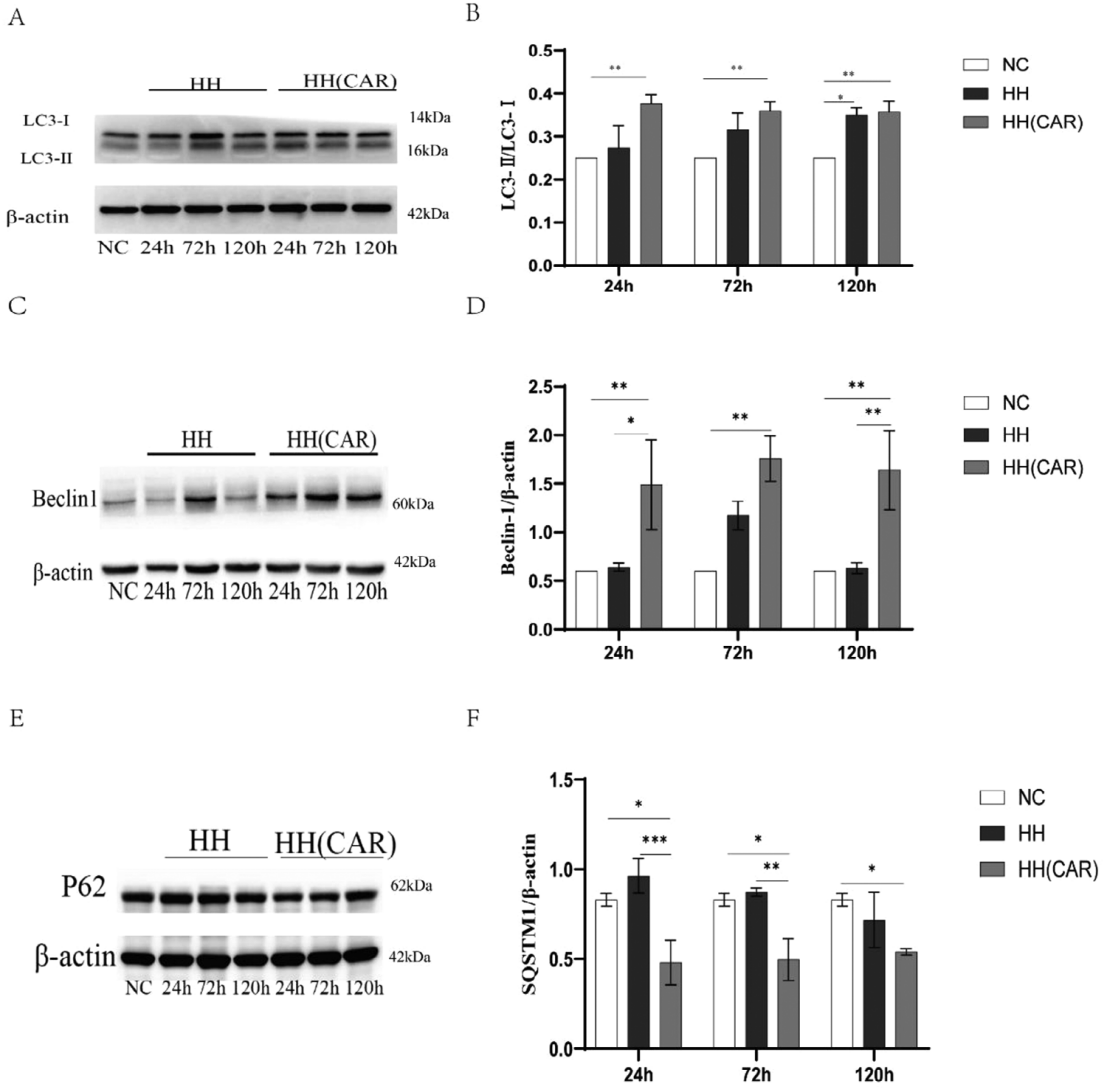
Expression of Autophagy‐Related Proteins in Rat Lung Tissue. Western blotting was used to detect the expression of Beclin‐1, LC3‐I, LC3‐II, and SQSTM1/P62 proteins. Representative immunoblots from each experimental group are shown: A) Expression of LC3‐I and LC3‐II proteins; C) Expression of Beclin‐1 protein; E) Expression of SQSTM1/P62 protein. The results are presented as B) the ratio of the intensity of the LC3‐II band to the intensity of the LC3‐I band, D) the ratio of the intensity of the Beclin‐1 band to the intensity of the β‐actin band, or F) the ratio of the intensity of the SQSTM1/P62 band to the intensity of the β‐actin band. NC (Normal Control) group, HH (High‐Altitude Hypoxia) group, and HH(CAR) (a group subjected to High‐Altitude Hypoxia after Controlled Ascent Rate). Data are presented as the mean ± SD (*n* = 10). ^*^ *p* <0.05, ^**^ *p *<0.01, ^***^ *p* <0.001.

As shown in **Figure** **6**, the occurrence of autophagy was assessed by detecting the expression of Beclin‐1, SQSTM1, and the ratio of LC 3‐II/LC 3 I using western blotting. Compared with the NC group, the expression of Beclin‐1 in the HH(CAR) group was significantly increased at each time point (*p* <0.01, Figure 6D). Compared with the NC group, the expression of Beclin‐1 in the HH group did not change significantly (*p* >0.05, Figure 6D). A higher LC 3‐II/LC 3‐I ratio indicates a higher level of autophagy. The ratio of LC 3‐II/LC 3‐I was analyzed (Figure 6A,B). Compared with the NC group, the LC 3‐II/LC 3‐I ratio in the HH(CAR) group was significantly increased at 24, 72, and 120 h (*p* <0.01, Figure 6B). At 120 h, the ratio of LC 3‐II/LC 3‐I in the HH group was also higher than that in the NC group (*p* <0.05, Figure 6B). Compared with the NC group, the expression of SQSTM1/P62 in the HH(CAR) group was significantly decreased at each time point (*p* <0.05, Figure 6F), while the expression of SQSTM1/P62 in the HH group was not significantly changed at each time point compared with the NC group (*p* >0.05, Figure 6F).

## 3 Discussion

High‐altitude regions constitute a natural hypoxic environment. Due to their unique characteristics of low pressure and thin oxygen, these environmental conditions can trigger various diseases. Acute hypoxia may cause respiratory discomfort, and in extreme cases, it can even lead to severe lung damage, pulmonary edema, and Acute Respiratory Distress Syndrome (ARDS).^[^ ^15^ ^]^ Pathological changes induced by high‐altitude environments are commonly associated with oxidative stress. This phenomenon can be substantiated by observing changes in specific biomarkers: an increase in MDA levels indicates enhanced oxidative damage, while a decrease in the activity of key antioxidant enzymes such as SOD and GSH‐Px further confirms the weakening of the body's antioxidant defense mechanisms.^[^ ^3^ ^]^ Our experimental results also confirmed this change. Hypoxia at high altitudes leads to an excessive production of ROS, which in turn triggers oxidative stress in the lungs of rats, ultimately resulting in ALI.^[^ ^16^, ^17^ ^]^ As the duration of hypoxic exposure extends, excessive oxidative stress can further stimulate the immune system and release a large accumulation of inflammatory factors within the body. Such outcomes may precipitate the occurrence of a cytokine storm, further damaging lung tissue and leading to Acute Mountain Sickness (AMS).^[^ ^18^ ^]^

Previous research has indicated that inhibiting oxidative stress, as well as the TLR4/NF‐κB/NLRP3 pathway, can ameliorate acute lung injury in rats exposed to high‐altitude hypoxia.^[^ ^19^ ^]^ Autophagy plays a role in a variety of human lung diseases, including pulmonary diseases such as chronic obstructive pulmonary disease (COPD), cystic fibrosis (CF), pulmonary arterial hypertension (PAH), and idiopathic pulmonary fibrosis (IPF).^[^ ^11^ ^]^ Meng et al.’s study indicates that interference with HIPK1 through siRNA can enhance autophagy, alleviate inflammation and oxidative stress in LPS‐induced acute lung injury in mice, and ultimately provide protective effects.^[^ ^20^ ^]^ The activation of autophagy is generally regarded as a protective mechanism for cells to combat oxidative stress.^[^ ^21^ ^]^

Research indicates that hypobaric hypoxia preconditioning (HHP) significantly mitigates the pulmonary edema, inflammatory responses, as well as ischemic and oxidative damage in the lungs induced by high‐altitude exposure (HAE).^[^ ^22^ ^]^ In fact, the most widely adopted strategy for preventing high‐altitude pulmonary edema is to ascend gradually to high altitudes, which is essentially a process of hypoxia acclimatization. By incrementally increasing altitude, the body can better adapt to the thin‐air environment, thereby reducing the risk of high‐altitude pulmonary edema. Under conditions of pulmonary hypoxia, the lung tissue may undergo local interstitial proliferation accompanied by bleeding. Within the alveolar cavities, there is an accumulation of a large number of red blood cells, along with an excessive infiltration of inflammatory cells. This infiltration of inflammatory cells may further exacerbate the damage and inflammatory response in the lungs.^[^ ^23^ ^]^ As demonstrated in this study, when animals are exposed to a simulated high‐altitude environment equivalent to 6000 meters above sea level in a hypobaric chamber, they exhibit varying degrees of interstitial edema, neutrophil infiltration, and hemorrhage depending on the duration of exposure. Rats that were allowed to ascend at a controlled rate exhibited mitigated pathological changes in lung tissue at various time points compared to the hypoxia stress group, characterized by mild interstitial edema and minimal infiltration of inflammatory cells. Studies have discovered that in animal models of idiopathic pulmonary fibrosis, hypoxia preconditioning can induce the expression of a range of survival factors, chemokines, and growth factors in pulmonary mesenchymal cells that are associated with cell proliferation, migration, angiogenesis, antioxidant activity, anti‐apoptosis, and anti‐fibrotic properties. The expression of these bioactive molecules contributes to enhancing the lung's self‐repair capabilities and optimizing the pulmonary tissue's response to and repair of injury.^[^ ^24^ ^]^

Our study found that rats controlled for ascent rates exhibited significant changes in oxidative stress levels compared to the hypoxia stress group. The results showed a decrease in MDA levels, indicating a reduction in oxidative damage, while the activities of two key antioxidant enzymes, SOD and GSH‐Px, increased. Chronic hypoxia preconditioning enhances the defensive capabilities of lung tissue against oxidative damage, a phenomenon confirmed in animal experiments.^[^ ^25^ ^]^ In the lung tissue, the activity of key antioxidant enzymes such as SOD and GSH‐Px is increased, which helps to reduce the level of oxidative stress, alleviate oxidative damage, and thereby protect lung tissue from damage caused by high‐altitude environments. Furthermore, our research discovered that the inflammatory levels in rats with controlled ascent velocities exhibited distinct changes compared to the hypoxia stress group, with the results demonstrating a significant reduction in the levels of the inflammatory factors IL‐6 and IL‐1β. Previous studies have indicated that hypoxic preconditioning, as a novel non‐pharmaceutical treatment for pulmonary inflammation, can effectively improve patient outcomes and recovery.^[^ ^26^ ^]^ Hypoxia preconditioning can effectively alleviate inflammation in alveolar epithelial cells by activating the HIF signaling pathway.^[^ ^27^ ^]^

Arterial blood gas (ABG) measurement is a key indicator for assessing the degree of pulmonary gas exchange abnormalities, which includes the determination of PO2, PCO2, and pH values in arterial blood.^[^ ^28^ ^]^ In high‐altitude environments, the changes in the ventilatory function of rats with ALI can be accurately evaluated through arterial blood gas analysis, which is crucial for determining whether specific interventions have a protective effect against lung injury. Arterial blood gas parameters are influenced by a multitude of factors, including the functionality of the respiratory and circulatory systems, cellular metabolism, renal regulatory actions, and the efficiency of alveolar ventilation.^[^ ^29^ ^]^ High‐altitude hypoxia triggers a series of physiological responses, among which the Hypoxic Ventilatory Response (HVR) is a crucial adaptive mechanism. HVR refers to the body's natural reaction to a low‐oxygen environment, increasing the frequency and depth of breathing to enhance oxygen intake, thereby partially alleviating the hypoxic state caused by low oxygen. This response can lead to changes in Arterial Blood Gas (ABG) parameters to adapt to the oxygen‐poor environment.^[^ ^30^ ^]^ The fact has proven that, in particular, the SpO2 levels decrease with acute high‐altitude/hypoxia exposure and partially recover during the acclimatization process.^[^ ^31^ ^]^ In our study, after exposure to a high‐altitude environment for 24, 72, and 120 h, the levels of SO2 and PO2 in rats both decreased. Our study also found that after exposure to a high‐altitude environment for 24 h, rats exhibited a decrease in PCO2 and an increase in blood pH, indicating that they experienced a hyperventilation response. This hyperventilation may activate the acid‐base compensatory mechanism in the kidneys.^[^ ^32^ ^]^ After exposure to a high‐altitude environment for 72 and 120 h, the SO2 and PO2 in rats remained low, while the blood pH gradually decreased and the PCO2 gradually increased. This phenomenon indicates that as the duration of high‐altitude exposure extends, the respiratory system of rats is significantly affected. PCO2 is a more sensitive indicator of respiratory failure than PO2 because it is closely related to the depth and frequency of breathing.^[^ ^33^ ^]^ The study also demonstrates that with prolonged exposure to high‐altitude environments, rats transition from initial hyperventilation to respiratory failure.^[^ ^34^ ^]^ The acid‐base balance within the rats' internal environment shifts from respiratory alkalosis to respiratory acidosis. Moreover, due to sustained hypobaric hypoxia, lactate accumulates progressively in the blood, leading to a significant increase in plasma lactate levels and exacerbating metabolic acidosis.^[^ ^35^ ^]^ In this study, after 120 h of exposure to high altitude, the lactate levels in rats were significantly higher compared to other groups, indicating that with extended exposure to high altitude, the rats' ability to regulate acid‐base balance becomes compromised. Rats treated with controlled ascent rates exhibited significantly lower PCO2 and higher pH at the same time points compared to the hypoxia stress group, with no significant differences in PCO2 and pH compared to the plain control group. These results suggest that rats treated with controlled ascent rates can enhance their pulmonary resistance to hypoxia stress. Consistent with our experimental findings, rats that underwent controlled ascent exhibited significantly reduced blood lactate levels at the 120 h time point compared to the hypoxia stress group. Previous studies have confirmed that there is a correlation between arterial blood lactate concentration and the severity of pulmonary injury.^[^ ^36^ ^]^ Therefore, the aforementioned blood gas analysis results indicate that rats subjected to controlled ascent can significantly mitigate high‐altitude hypoxia‐induced pulmonary injury in a high‐altitude environment.

Our research findings indicate that rats controlled for ascent rate and subsequently exposed to a high‐altitude hypoxic environment show a significant increase in Beclin‐1 expression, enhanced conversion of LC3‐I to LC3‐II, and a marked decrease in SQSTM1/P62 expression. These results suggest that rats treated with a controlled ascent rate can significantly promote autophagy in a high‐altitude hypoxic environment.^[^ ^37^ ^]^ Following autophagy activation, pathological changes in lung tissue, lipid peroxidation, and inflammatory responses are all alleviated compared to rats rapidly exposed to high‐altitude conditions. Current research indicates that autophagy, as an intracellular degradation and recycling mechanism, can alleviate inflammatory responses by regulating the production of inflammatory factors. Specifically, autophagy is capable of reducing the secretion levels of inflammatory cytokines, thereby effectively improving ALI induced by lipopolysaccharide (LPS).^[^ ^38^ ^]^ The activation of autophagy plays a crucial role in the pathophysiological processes of sepsis, as it helps to mitigate the excessive release of cytokines and pulmonary damage caused by sepsis. By facilitating the selective degradation of damaged cellular organelles and inflammatory mediators, the process of autophagy aids in maintaining cellular homeostasis and preventing uncontrolled inflammatory responses.^[^ ^39^ ^]^ Additionally, studies have found that activating autophagy can promote the nuclear translocation of Nrf2, enhance the expression of antioxidant factors such as heme oxygenase‐1(HO‐1) and NAD(P)H quinone dehydrogenase 1 (NQO1), restore the activity of antioxidant enzymes, reduce the accumulation of ROS, and alleviate oxidative stress.^[^ ^40^ ^]^ Our research findings indicate that the activation of autophagy reduces the levels of IL‐6, IL‐1β, and MDA while simultaneously enhancing the activities of GSH‐Px and SOD. Upon comprehensive analysis, autophagy appears to mitigate pulmonary injury induced by high‐altitude hypobaric hypoxia by suppressing oxidative stress and inflammatory responses.

## 4 Conclusion

In summary, this study demonstrates that a high‐altitude hypobaric hypoxic environment induces ALI in rats by promoting oxidative stress and inflammation. Rats that ascend gradually and are then exposed to a high‐altitude hypobaric hypoxic environment are more capable of modulating the activation of autophagy. Autophagy plays a protective role in ALI induced by high‐altitude hypobaric hypoxia by alleviating oxidative stress and inflammatory responses. However, it should be noted that these observations were obtained in animal models and cannot be directly extrapolated to the development of AMS, HAPE, and high‐altitude cerebral edema (HACE) in humans. Future research needs to further explore the roles of these mechanisms in human high‐altitude diseases. In addition, controlling the ascent rate to mitigate ALI in high‐altitude hypoxic conditions involves a complex interplay of physiological and biological pathways. Our research on the protective mechanisms against high‐altitude ALI has predominantly concentrated on the level of autophagy‐related proteins. Despite this focus, the current comprehension of these protective mechanisms remains limited and is in need of more direct evidence. Future investigations should employ more intuitive detection methods, such as electron microscopy (EM), to observe alterations in intracellular structures, thus providing more concrete evidence. Further research is essential to explore the unknown mechanisms in this domain. By integrating EM detection, genomic testing, and assessments at the protein level, we anticipate achieving a more holistic and profound understanding of these protective mechanisms in the future.

## 5 Experimental Section

### Animals and Ethics

A total of 70 Sprague–Dawley (SD) male rats weighing from 220 to 260 g, were purchased from the Experimental Animal Center of Xinjiang Medical University (Urumqi, China). The rats were kept under the specific pathogen‐free animal laboratory at the Key Laboratory of Special Environmental Medicine of Xinjiang, General Hospital of Xinjiang Military Command (license number: SYXK (military) 2017‐0050), at least one week prior to the experiments, the animals were housed under conditions of 23–25 °C with free access to food and water, and maintained on a 12 h light/12 h dark cycle. This study has been approved by the Institutional Animal Care and Use Committee of the General Hospital of Xinjiang Military Command (Approval No.: DWLL 2023102202), and it strictly adheres to the institution's guidelines for the care and use of laboratory animals. To minimize the potential confounding effects of gender differences on the experimental outcomes, this study wasintented to experiment exclusively with male rats.

### Experimental Methods

Using the method of random number tables, 70 SD rats were divided into 7 groups (10 rats per group): a Normal Control (NC) group, a High‐Altitude Hypoxia 24 h(HH24) group, a High‐Altitude Hypoxia 72 h (HH72)group, a High‐Altitude Hypoxia 120 h (HH120) group, a group subjected to High‐Altitude Hypoxia 24 h after Controlled Ascent Rate(HH24CAR), a group subjected to High‐Altitude Hypoxia 72 h after Controlled Ascent Rate(HH72CAR), and a group subjected to High‐Altitude Hypoxia 120 h after Controlled Ascent Rate(HH120CAR). The rats in the NC group were housed in a standard temperature environment with temperatures controlled within a range of 23 °C (±2 °C) and relative humidity maintained at 45% (±5%). The remaining groups of rats were placed in a simulated high‐altitude environment within Simulated Climate Cabin for Special Environments in Northwest China (Urumqi, China). The environmental conditions within the experimental chamber were as follows: a 12 h light/12 h dark cycle, with a continuous 12 h at 15 °C during the day and 12 h at 5 °C during the night, along with a relative humidity of 45% (±5%). Rats in the NC group were exposed to a plain environment, whereas rats in the high‐altitude hypoxia 24 h group, high‐altitude hypoxia 72 h group, and high‐altitude hypoxia 120 h group were exposed to the conditions within the experimental chamber. The simulated altitude of the chamber was set to 6000 meters, with an ascent rate of 10 meters per second. Upon reaching the designated altitude, the chamber maintained a humidity level of 45% (±5%). The three groups that controlled the ascent rate followed the recommendations of the Wilderness Medical Society. Initially, they were set at an altitude of 2000 meters and ascended continuously for three days, increasing the altitude by 500 meters each day. They then stayed at an altitude of 3500 meters for one day. On the fifth day, they continued to ascend, maintaining the pattern of ascending 500 meters each day for three more days, before staying at an altitude of 5000 meters for another day. Finally, they ascended for two consecutive days, increasing the altitude by 500 meters each day, until they reached 6000 meters, at which point the ascent was halted^1^. When the device reached the set altitude, the humidity was maintained at 45% (±5%). The groups that controlled the ascent rate were then exposed to an altitude of 6000 meters for 24, 72, and 120 h, respectively, according to the predetermined schedule. Upon reaching the designated exposure durations, rats were sequentially extracted from the experimental chamber according to the scheduled time points and promptly anesthetized. All rats were anesthetized via intraperitoneal injection of 2% sodium pentobarbital at a dosage of 2 mL per 0.1 kg of body weight. Post‐anesthesia, the rats were positioned supine, and a midline longitudinal incision was made in the abdominal wall to fully expose the aorta. Arterial blood was collected for subsequent blood gas analysis. Following euthanasia, the thoracic cavity was opened to harvest lung tissues, which were then dissected into distinct lobes for further detailed analysis. The harvested tissues were utilized for the determination of the wet‐to‐dry weight ratio (W/D), histological examination, enzyme‐linked immunosorbent assay (ELISA), and Western blot analysis.

### Arterial Blood Gas Analysis and Plasma Lactate Measurement

After arterial blood collection, the ABG levels were immediately measured using the i‐STATm blood gas analyzer (Abbott Laboratories, Princeton, NJ, USA).

### Lung Wet/Dry Weight Ratio (W/D)

After the humane euthanasia of the rats, the right lower lobe of the lung was excised, and any blood contamination on the surface of the lung tissue was gently absorbed with filter paper before placing it on aluminum foil. The wet weight was immediately measured using an analytical balance. The lung tissue, along with the aluminum foil, was then placed in an electric oven at 80 °C and dried to a constant weight for 72 h. The dry weight was obtained by weighing again with the analytical balance. The wet‐to‐dry (W/D) weight ratio, which serves as an indicator of pulmonary edema, was calculated using the following formula: Lung wet weight to dry weight ratio = wet weight / dry weight.

### Histologic Assessment

Collect the right middle lobe of the rat's lung, thoroughly wash it with physiological saline, and then fix it with a 4% paraformaldehyde solution. Subsequently, perform Hematoxylin and Eosin (H&E) staining on the lung tissue samples for pathological examination. After staining, observe the lung tissue samples under an optical microscope to identify any pathological changes. The assessment of lung injury was conducted using a standardized scoring system that includes three criteria: 1) the presence or absence of edema; 2) the degree of neutrophil infiltration; and 3) the severity of congestion. The severity of lung injury was graded as follows: no injury was scored 0; injury affecting less than 25% of the lung field was scored 1; injury affecting 50% of the lung field was scored 2; injury affecting 75% of the lung field was scored 3; and injury affecting the entire lung field was scored 4.^[^ ^41^ ^]^

### Determination of Inflammatory Cytokines and Oxidative Stress Levels in Lung Tissue

Wash the left lung lobe tissue samples in pre‐cooled phosphate‐buffered saline (PBS) to remove blood and impurities. Take a portion of the cleaned left lung lobe tissue samples and cut them into small pieces, adding lysis buffer (Solarbio Technology Co., Ltd, Beijing, China) at a ratio of 200 µL per 20 mg of tissue. A low‐temperature grinder (Wuhan Servicebio Technology Co, Ltd, China) was used for grinding. After grinding, centrifuge the homogenate at low temperature (4 °C) at high speed (12000 × g for 5 min), and then take the supernatant for ELISA experiments. Quantify the protein concentration in the supernatant using the BCA method and according to the instructions of the enzyme‐linked immunosorbent assay (ELISA) kits (Shanghai FANKEL Industrial Co., Ltd., China), respectively determine the levels of interleukin IL‐1β (Interleukin‐1β), IL‐6 (Interleukin‐6), glutathione peroxidase (GSH‐PX), malondialdehyde (MDA), and superoxide dismutase (SOD) in the supernatant.

### Western Blotting

A portion of the left lung lobe was lysed in ice‐cold RIPA buffer (Solarbio Technology Co., Ltd, Beijing), with the addition of a protease inhibitor cocktail. The tissue lysates were centrifuged at 12 000 g for 5 min at 4 °C, and the supernatants were collected. Total protein concentrations were determined using a BCA protein assay kit (Solarbio Technology Co., Ltd, Beijing). One hundred micrograms of protein extracts from each sample were used for Western blot analysis. Proteins were separated by sodium dodecyl sulfate‐polyacrylamide gel electrophoresis (SDS‐PAGE) on 12% polyacrylamide gels, according to the required molecular weight separation range. After transferring the proteins to nitrocellulose membranes (Solarbio Technology Co., Ltd, Beijing), the membranes were blocked with 5% skim milk in TBST (Tris‐buffered saline with 0.1% Tween 20) at 37 °C for 2 h. The primary antibodies used were: anti‐Beclin‐1 (1:1000, Cell Signaling Technology, Danvers, MA, USA), anti‐LC3‐I/II (1:1000, Cell Signaling Technology, Danvers, MA, USA), anti‐SQSTM1/P62 (1:1000, Cell Signaling Technology, Danvers, MA, USA), and anti‐β‐actin (1:1000, Cell Signaling Technology, Danvers, MA, USA). After washing, the membranes were incubated with horseradish peroxidase (HRP)‐conjugated secondary antibodies (Wuhan Sanying Biotechnology Co., Ltd). Protein bands were visualized using a Bio‐Rad imaging system (Bio‐Rad Laboratories, Inc., California, USA), and densitometric analysis was performed using Image J software. The results were presented as the ratio of the intensity of Beclin‐1 and SQSTM1/P62 bands to that of β‐actin, or the ratio of the intensity of the LC3‐II band to that of the LC3‐I band.

### Statistical Analysis

Data were presented as the mean ± standard deviation. Statistical analysis was performed using SPSS software version 27.0 (Chicago, IL, USA). Graph generation was conducted with GraphPad Prism software version 10.1.2 (San Diego, CA, USA). For comparing differences among multiple groups, one‐way ANOVA followed by LSD post‐hoc tests were employed. In all experiments, a p‐value less than 0.05 was considered to indicate statistical significance, and a p‐value less than 0.01 was considered to indicate highly significant statistical significance.

## Conflict of Interest

The authors declare no conflict of interest.

## Data Availability

The data that support the findings of this study are available from the corresponding author upon reasonable request.
